# Effects of MGC-0109, a slow-release sulphide donor, on myocardial ischaemia/reperfusion injury

**DOI:** 10.1186/2197-425X-3-S1-A957

**Published:** 2015-10-01

**Authors:** A Dyson, A Lach, M Singer

**Affiliations:** Magnus Oxygen Ltd, London, United Kingdom; University College London, London, United Kingdom

## Introduction

MGC-0109 (ammonium tetrathiomolybdate) is a long-acting sulphide donor that reversibly inhibits mitochondrial respiration. We previously found that MGC-0109 reduced oxidative stress and doubled 6-hour survival in a severe haemorrhage/reperfusion rat model (1). Here we explore its utility in an organ-specific ischaemia/reperfusion injury.

## Objectives

To assess the efficacy of MGC-0109 in a rat model of myocardial ischaemia/reperfusion injury.

## Methods

Ventilated, anaesthetized rats (4/group) were instrumented with vascular lines for drug/fluid administration. Following thoracotomy, the left anterior descending (LAD) coronary artery was ligated for 30 min. Just prior to reperfusion, animals received i.v. MGC-0109 (10 mg/kg bolus) or n-saline. MGC-0109-treated animals received a further 2.5 mg/kg infusion over 15 min. Control animals received equivalent volumes of n-saline. Animals were monitored for a further 4 h. The LAD was then re-ligated and perfused with Evans Blue dye to determine the area at risk. Hearts were then excised, sliced in 1mm sections distal to the LAD ligature, and incubated with (red) tetrazolium chloride (taken up by non-infarcted tissue). Statistics: Non-parametric t test.

## Results

MGC-0109 given at reperfusion reduced infarct size by 40%. Due to the current small sample size, these values are not statistically significant.

## Conclusions

MGC-0109 given just prior to reperfusion shows protective utility in myocardial ischaemia/reperfusion injury.

## Grant Acknowledgment

Funded by Magnus Oxygen.

**Figure 1:**
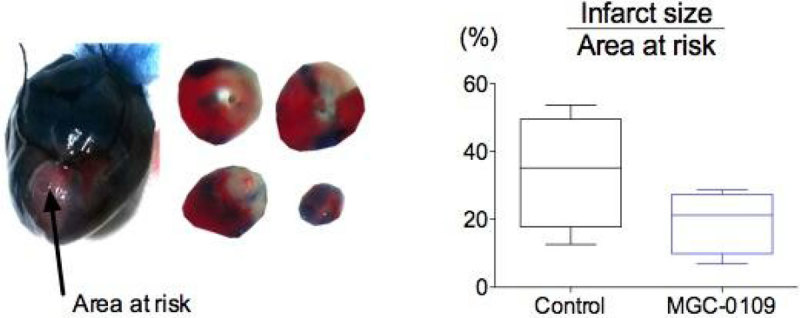
Figure 1
